# Mussel parasite richness and risk of extinction

**DOI:** 10.1111/cobi.13979

**Published:** 2022-09-26

**Authors:** Joshua I. Brian, David C. Aldridge

**Affiliations:** ^1^ Department of Zoology University of Cambridge Cambridge UK; ^2^ Department of Geography King's College London London UK; ^3^ BioRISC, St Catharine's College Cambridge UK

**Keywords:** ciliate, coextinction, mite, richness, trematode, unionid, Ácaro, ciliado, coextinción, trematodo, uniónido

## Abstract

Parasite conservation is important for the maintenance of ecosystem diversity and function. Conserving parasites relies first on understanding parasite biodiversity and second on estimating the extinction risk to that biodiversity. Although steps have been taken independently in both these areas, previous studies have overwhelmingly focused on helminths in vertebrate hosts over broad scales, providing low resolution and excluding a large proportion of possible host and parasite diversity. We estimated both total obligate parasite richness and parasite extinction risk in freshwater mussels (Unionidae and Margaritiferidae) from Europe and the United States to provide a case study for considering parasite conservation in a severely understudied system. We used currently reported host–parasite relationships to extrapolate parasite diversity to all possible mussel hosts and then used the threat levels of those hosts to estimate the extinction risk for both described and undescribed parasites. An estimated 67% of parasite richness in freshwater mussels is undescribed and over 80% of the most host‐specific groups (digenean trematodes and ciliates) are undescribed. We estimated that 21% of this total parasite fauna is at immediate risk of extinction, corresponding to 60 unique species, many of which will likely go extinct before being described. Given the important roles parasites play in community structure and function and the strong ecosystem engineering capacities of freshwater mussels, such extinctions are likely to severely affect freshwater ecosystems. Our detailed study of mussel parasites provides compelling evidence for the hidden conservation threat to parasites through extinction cascades and shows parasites are deserving of immediate attention.

## Abstract 2

*Article Impact Statement*: Two‐thirds of freshwater mussel parasites are undescribed and over one‐fifth are at immediate risk of extinction.

## Resumen

La conservación de parásitos es importante para el mantenimiento de la diversidad y funcionamiento de los ecosistemas. La conservación de parásitos depende en primera instancia del entendimiento de la biodiversidad de parásitos y, en segunda, de la estimación del riesgo de extinción de esa biodiversidad. Mientras que se han tomado medidas en ambas áreas, estudios previos se han enfocado abrumadoramente en helmintos de hospederos vertebrados, proporcionando baja resolución y excluyendo a una amplia proporción de una probable diversidad de hospederos y parásitos. Estimamos tanto la riqueza de parásitos obligados como el riesgo de extinción de mejillones de agua dulce (Unionidae y Margaritiferidae) de Europa y Estados Unidos para proporcionar un caso de estudio para considerar la conservación de parásitos en un sistema severamente poco estudiado. Utilizamos las relaciones hospedero‐parásito registradas hasta la fecha para extrapolar la diversidad de parásitos a todas las especies posibles de mejillones hospederos y luego utilizamos los niveles de amenaza de aquellos hospederos para estimar el riesgo de extinción tanto para parásitos descritos y no descritos. Alrededor de 67% de la riqueza de parásitos de mejillones de agua dulce no esta descrito, así como mas de 80% de los grupos específicos de hospederos (trematodos digeneos y ciliados) tampoco están descritos. Estimamos que 21% del total de esta fauna de parásitos está en riesgo inminente de extinción, correspondiendo a 60 especies únicas, muchas de las cuales probablemente se extinguirán antes de ser descritas. Dado la importancia del papel que desempeñan los parásitos en la estructura y función de la comunidad y las notables capacidades de los mejillones de agua dulce para la ingeniería del ecosistema, es muy probable que tales extinciones afecten severamente a los ecosistemas dulceacuícolas. Nuestro estudio detallado de los parásitos de mejillones proporciona evidencia convincente de la amenaza oculta para los parásitos mediante cascadas de extinción y muestra que los parásitos son merecedores de atención inmediata.

## INTRODUCTION

Parasitism is a common and ecologically important evolutionary strategy; parasites often play a central role in the functioning of ecosystems (Hudson et al., 2006). Parasites can exert direct influence by generating an “extended phenotype” through parasitized organisms (Dawkins, 1982) and as free‐living biomass in transmission stages that provide an important but often overlooked food source (McKee et al., 2020; Mironova et al., 2020; Morley, 2012). Through their regulation of host populations (Tompkins & Begon, 1999), they encourage the coexistence of multiple species (Strona, 2015). However, parasites can also have significant negative effects at the individual, population, and community levels, and in extreme cases can lead to extirpation (Katsanevakis et al., 2019) or extinction of species (Daszak et al., 2000). Despite the important roles parasites play, their diversity remains poorly understood (Okamura et al., 2018). However, recent attempts have been made to estimate the diversity of some parasite groups, with a particular emphasis on vertebrate helminths (Carlson et al., 2020a; Dobson et al., 2008).

In addition to their diversity, the risk of extinction in parasite species has been underappreciated (Carlson et al., 2017). In this era of global change, communities are being assembled and dissembled at increasing rates (Pandolfi et al., 2020). There is strong evidence that parasite communities are no exception. Some parasite species are spreading unpredictably (Gillis‐Germitsch et al., 2020), whereas populations of other species have collapsed (Sitko & Heneberg, 2020). Indeed, the extinction risk for parasites is becoming increasingly appreciated (Carlson et al., 2020b; Strona, 2015), and they may be the most at‐risk group of organisms on Earth (Dunn et al., 2009). Specialist parasites that rely on threatened hosts are particularly at risk, and there is clear evidence that coextinctions have already taken place (e.g., Boast et al., 2018). Further, extinction risk in parasites may be severely underestimated given a large majority of parasites are yet to be described (Carlson et al., 2020a). This risk may be particularly high for obligate parasites of molluscs, a phylum with extinction rates far higher than most taxa (Cowie et al. 2022).

One such threatened mollusc group with poorly described parasite diversity is freshwater mussels of the families Unionidae and Margaritiferidae (Brian & Aldridge, 2019) (henceforth freshwater mussels). Freshwater mussels provide important ecosystem services in global freshwater ecosystems (Vaughn, 2018), yet a large proportion of them are at risk of extinction (Lopes‐Lima et al., 2018). Freshwater mussels host a broad range of parasites (Brian & Aldridge, 2019), but the diversity (and corresponding risk of extinction) and impact of these parasites is poorly understood (Brian & Aldridge, 2019; Ferreira‐Rodriguez et al., 2019; Grizzle & Brunner, 2009). Therefore, we aimed (SSG to estimate the total diversity of parasites in freshwater mussels in the United States and Europe (the two best‐studied regions [Lopes‐Lima et al., 2018]) and to predict, based on this measure of total diversity, the proportion of parasites, described and undescribed, at risk of extinction. Previous researchers considered parasite diversity or extinction risk (e.g., Carlson et al., 2020a; Dobson et al., 2008; Farrell et al., 2015; Koh et al., 2004), but not both (i.e., estimates of extinction risk have been based only on described parasites). This knowledge gap means that parasite species could potentially go extinct before they have even been described (Lees & Pimm, 2015). Further, studies have been biased toward certain parasite groups (e.g., helminths). We focused on a severely understudied host group (freshwater mussels) and considered the full‐known parasite community rather than a specific taxonomic subset.

## METHODS

The parasites of freshwater mussels in the United States and Europe were chosen because these are the most well‐studied areas for freshwater mussels and they were the subject of a recent comprehensive review that lists all recorded host–parasite interactions in these regions (Brian & Aldridge, 2019). This review recorded every organism reported inside a freshwater mussel (i.e., endosymbionts) from the 279 extant U.S. native mussel species and 16 extant European native mussel species (according to the lists of Williams et al. [2017] and Lopes‐Lima et al. [2017]). For the full data set, see Tables A2 and A3 in Brian and Aldridge (2019).

To make estimates as reliable as possible, we removed records if the endosymbiont was not recorded to species level. To make interpretations of parasite extinction valid, we removed records of endosymbionts that could not be considered obligate parasites (i.e., we excluded macroinvertebrates, nematodes, bacteria, and viruses that may be incidental recordings). This left 96 unique parasite species observed inside the 295 possible host mussel species, although only 128 mussel species had records. In total, the data set consisted of 485 unique host–parasite combinations. These are in Appendices S3–S6.

To understand the relationship between host and parasite diversity, we first calculated the total number of parasite species observed in the United States and Europe. We then calculated the average number of parasite species present in a given host species and tested to see whether there was a difference between the U.S. and European mussel species with a Kruskal–Wallis test. This represents a host‐centric view (i.e., the number of parasite species a host species has).

We also took a parasite‐centric view (i.e., the number of host species a parasite species has). We classified the 96 parasite species into four main groups: aspidogastrean trematodes, digenean trematodes, mites, and ciliates. Given the different life‐history strategies displayed by these four groups (see Brian & Aldridge, 2021), they might show different levels of host generalism, which would need to be accounted for when predicting total parasite diversity. We tested this by comparing the median host range (number of different mussel species a parasite has been found in) among the four groups with a Kruskal–Wallis test. Given the difference in the total number of possible hosts between the United States and Europe, this was carried out separately for each region.

**FIGURE 1:**
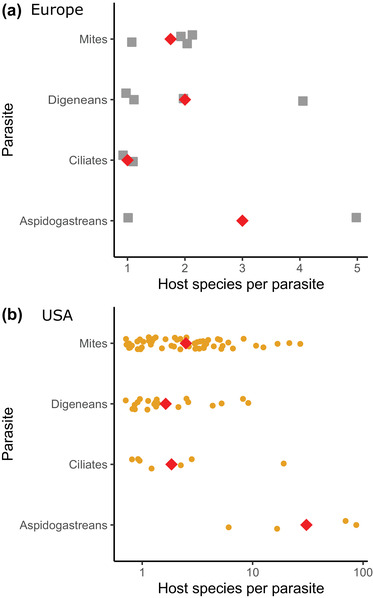
(a) Number of host species for parasites in the four parasite groups in (a) European and (b) U.S. mussels (points, individual parasite species [jittered to aid visualization]; red diamonds, overall mean). Graphs have different x‐axis scales (Europe, linear; the United States, log) given the much higher number of possible total hosts in the United States

Because parasites did display different levels of host specificity (see Results) (Figure 1), the following analyses were carried out separately for aspidogastrean trematodes, digenean trematodes, mites, and ciliates. To explore how parasite diversity scaled with host diversity in our four parasite groups, we considered a recent theory that contends parasite diversity does not scale linearly with host diversity but follows a power law because this appropriately accounts for hosts sharing generalist parasites (Carlson et al., 2019; Strona & Fattorini, 2014). Specifically, the function is

(1) $$\mathrm{parasites}\propto b^{*}{\mathrm{hosts}}^{z},$$

where *b* and *z* are parameters to be estimated that determine the shape of the curve. First, for each parasite group, we fitted this function to our observed data (i.e., to the 128 mussel species with records) to find *b* and *z*. We combined U.S. and European records to calculate total estimates of parasite diversity because there was no evidence parasite species richness accumulated differently on a per host basis between the two regions (see Results). Then, using our defined curves (Table 1), we extrapolated up to all 295 possible host mussel species and thus gained an estimate for the total number of parasites expected to be hosted by freshwater mussels in the United States and Europe. For both steps, we used the codependent package (Carlson, 2019) in R 3.6.3 (R Core Team, 2020).

**TABLE 1:** Power law functions describing how parasite diversity scales with host diversity for the four parasite groups of freshwater mussels

Parasite group	Function (general equation: parasites ∝ b*hosts*^z^*)
Aspidogastrean trematodes	*P* ∝ 2.31*H* ^0.17^
Digenean trematodes	*P* ∝ 2.54*H* ^0.69^
Mites	*P* ∝ 5.62*H* ^0.53^
Ciliates	*P* ∝ 1.25*H* ^0.62^

Finally, using our total estimated diversity of freshwater mussel parasites, we predicted how parasite extinction risk is related to host extinction risk. We first plotted the proportion of parasites expected to go extinct for a certain proportion of hosts going extinct with Equation (1). We assumed that hosts are lost in random order (following Koh et al., 2004). Although it would be more realistic to weight host extinction order according to current threat risk, it would be uninformative to do so in the present circumstance because, with our scaling up to total estimated parasite diversity, we were operating under the implicit assumption that threatened and nonthreatened hosts accumulate parasites at the same rate. In other words, we assumed the same rates of generalism or specificity in threatened and nonthreatened hosts. Despite very low sampling of endangered species (Brian & Aldridge, 2019), this assumption appears sound (Appendix S1). (See Discussion for explorations of our underlying assumptions and the conservativeness of our estimates.) Operating under this assumption, we estimated how many parasites would go extinct if all endangered and threatened mussel hosts went extinct. We provided estimates for each parasite group separately, as well as overall. To estimate host threat status, we used the U.S. Fish and Wildlife Service's Endangered Species list, which lists species as either threatened (TH), endangered (EN), or not listed and is the most reliable source for U.S. freshwater mussels. Because European species are not listed in this database, we used the IUCN Red List for freshwater mussels from Europe and translated the results into the same categories as mussels on the U.S. list, whereby critically endangered and endangered European mussels were classified as EN and vulnerable mussels were classed as TH. We also repeated the entire analysis with the IUCN categories for both U.S. and European mussels. These results were highly similar, so we present the results of the IUCN analysis in Appendix S1.

## RESULTS

### Parasite specificity and diversity in freshwater mussels

In total, there were 12 different parasite species in European mussels and 84 different parasite species in the U.S. mussels. In terms of parasite richness per host species, there was no difference between the United States and Europe (χ^2^_1_ = 0.88, *p* = 0.346); mussel species in both locations had a similar mean number of parasite species (Europe, 4.40 [SE 0.96]; the United States, 3.76 [0.26]). This suggests that the much greater parasite diversity in the United States is driven by the higher total number of mussel species, rather than a higher parasite richness per mussel species.

The four parasite groups differed in terms of their host specificity in Europe (Figure 1a) and the United States (Figure 1b). The statistical significance of this difference could not be assessed for Europe given low total parasite richness (*n* = 2 species for both aspidogastreans and ciliates), but this difference was significant for the United States (χ^2^_3_ = 14.63, *p* = 0.002). In both locations, aspidogastrean trematodes were the most host generalist; mites, digenean trematodes, and ciliates all showed high host specificity. In the United States, the median number of host species per parasite species was 1 for ciliates and digenean trematodes and 2 for mites.

**FIGURE 2:**
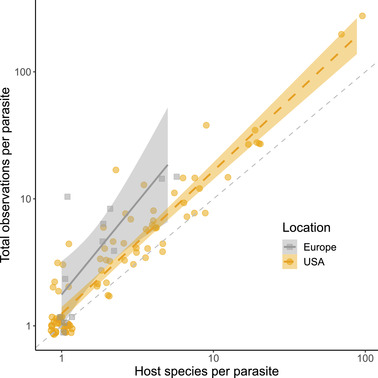
Total number of observations for each parasite (across all mussel hosts) correlated with the number of host species in which the parasite has been observed (shading, 95% CI of the fitted line). The line of isometry (dashed gray line) represents a 1:1 relationship between the host range and the total number of observations (e.g., a point lying on this line at [2, 2] or [10, 10] indicates a parasite has been observed once each in 2 or 10 hosts, respectively)

High host specificity may have been overestimated given that most hosts were underdocumented. There was a strong correlation between host range and the total number of observations of each parasite (Figure 2), indicating that the more a parasite was observed, the more hosts it was found in. (Thus, if we had sampled more host mussel species, we would have found the same parasites repeated in those hosts.) The 95% CIs for the United States and Europe did not overlap the line of isometry at any point, indicating most parasites were observed multiple times in certain host species and were, therefore, reliably absent from at least some other host mussels. These parallel yet vertically separated lines also demonstrated that the parasites of European mussels had been proportionally better documented than mussels in the United States, but that parasites likely accumulated in a consistent fashion between the regions. The cluster of points at the bottom left of Figure 2 corresponds to parasites observed once in a single host species. In this case, it was impossible to tell whether they were rare host‐specific parasites or cryptic (to date) generalists that were overlooked in other hosts.

We estimated that 67% of parasite diversity in the U.S. and European mussels is yet to be described, including roughly 80% of all digenean trematodes and ciliates (Table 2). In contrast, given their highly host‐generalist nature (Figure 1a,b), we predicted that nearly all aspidogastrean diversity had been observed, with possibly one more species yet to be described.

**TABLE 2:** Currently observed and predicted^a^ total number of parasite species of 295 mussels of Europe and the United States

Parasite group	Observed number of species	Predicted number of species (95% CI)	Undescribed parasites (%)
Aspidogastrean trematodes	5	6 (6–6)	16
Digenean trematodes	25	127 (116–138)	80
Mites	58	115 (111–118)	49
Ciliates	8	42 (36–50)	81
Total	96	289 (268–312)	67

^a^ Rounded to the nearest whole number.

### Risk of parasite extinction

**FIGURE 3:**
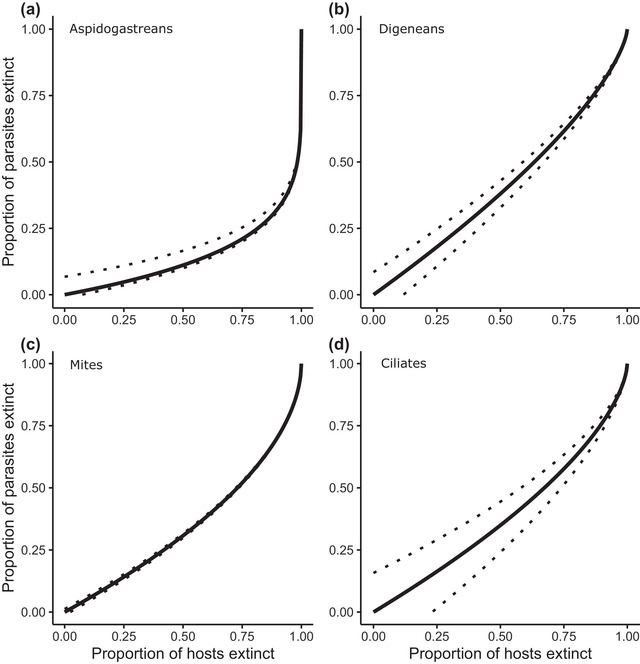
Predicted proportion of parasites that would go extinct for a given proportion of mussel hosts going extinct (solid lines, mean estimates; dotted lines, 95% CIs): (a) aspidogastreans, (b) digeneans, (c) mites, and (d) ciliates

In all cases (but to varying degrees), estimating the threatened nature of described and undescribed parasites showed that parasite extinction risk was initially low relative to the host extinction rate, but then quickly rose as more hosts become extinct, due to the reduced ability of generalist parasites to host‐switch at lower total host diversity (Figure 3).

Threatened host species did not have fewer specialist parasite species (Appendix S1); hence, we found that host and parasite endangerment risks were directly related. When we allowed all threatened mussels to go extinct (EN + TH), we predicted that 21% of all U.S. and European freshwater mussel parasites would be lost (approximately 60 species) (Table 3). In the case of digenean trematodes and ciliates, this corresponded to more species going extinct than have been described. Even when we considered that only endangered mussels went extinct, we predicted the extinction of 47 parasite species. These estimates of mussel threat risk excluded data‐deficient mussel species or those that have not been assessed (in the case of IUCN estimates, this corresponded to 31% of all host species), so the proportion of mussels (and hence parasites) that were threatened would likely be higher than we predicted. The overall results are very similar when threat risk was assessed using the IUCN designations rather than the U.S. Fish and Wildlife Service designations (Appendix S1).

**TABLE 3:** Percentage of parasite species predicted to go extinct if all endangered (EN) mussel hosts go extinct or if all endangered and threatened (EN + TH) hosts go extinct

Hosts extinct	Aspidogastreans extinct (%)	Digeneans extinct (%)	Mites extinct (%)	Ciliates extinct (%)	Total parasites extinct (%)
EN	5.0	18.8	14.8	17.1	16.1
EN + TH	6.5	23.8	18.8	21.7	20.6

## DISCUSSION

### Estimates of parasite diversity and extinction risk

We estimated that nearly 70% of obligate freshwater mussel parasites are yet to be described and that over one‐fifth of these are at immediate risk of extinction. These results affirm the lack of knowledge surrounding parasites of freshwater mussels (Brian & Aldridge, 2019; Grizzle & Brunner, 2009) and the risks faced by parasite species globally (Carlson et al., 2017, 2020b). Because we extrapolated from an incomplete data set, there are inevitable sources of bias that could alter our conclusions. We identified at least nine sources of bias, seven of which would result in an underestimate of parasite diversity and extinction risk. Thus, we considered our estimates conservative.

Two sources of bias would result in an overestimate of richness and extinction risk. First, the power function used to extrapolate total species richness (Equation 1) is prone to slight overestimation (Carlson et al., 2019). However, it remains the most suitable curve for using host richness to estimate parasite richness and is relevant for a wide variety of systems, including human viruses, vertebrate trematodes, mammalian nematodes, and plant pollinators (Carlson et al., 2019, 2020a). Second, given the undersampling present in the mussel‐parasite system (Brian & Aldridge, 2019) (Figure 2), it is likely that parasites in the data set have associations with more host species than have currently been recorded (“missing links” [Dallas et al., 2017; Farrell et al., 2022]). This would cause an overestimation of host specificity and make the estimated curve steeper (i.e., increase the value of *z* in Equation 1), which would lead to unduly high estimates of richness. Further, the ability of the parasites to switch hosts (Hoberg & Brooks, 2008) to previously unoccupied hosts or hosts to be unoccupied due to incomplete sampling would also reduce extinction risk.

Four sources of bias that would lead to underestimation of diversity and extinction risk concern parasite sampling. First, while poor sampling coverage could lead to parasites already in the data set being incorrectly classified as absent from true hosts, it could also lead to a failure to observe new parasites in certain hosts (e.g., Carlson et al., 2020a). This would lead to an underestimate of host specificity (i.e., decrease *z*) and to unduly low estimates of diversity and extinction risk. Second, unsampled parasite species (those yet to be described), by virtue of their not being observed yet, are likely to be more host specialized than those that have been observed (Carlson et al., 2020a). Therefore, the undescribed proportion of parasite diversity will be more host‐specific: this has the same effect as the previous point and elevates the risk of extinction relative to currently described (more generalist) parasites. Third, there are likely to be many cryptic parasite species that are morphologically indistinguishable (Dobson et al., 2008). For example, *Aspidogaster conchicola* occurs in 92 mussel species, but it is highly unlikely that this is a single species (Alves et al., 2015). Indeed, trematodes have especially high rates of cryptic diversity (de León & Poulin, 2018). Fourth, our estimates of extinction risk could not account for species without sufficient data (e.g., 31% of the mussels on the IUCN Red List). It is likely that many of these species are also highly threatened (Lopes‐Lima et al., 2018), which means that our estimates of extinction risk should be higher. Indeed, mollusc extinction rates generally are severely underestimated and far higher than those of other taxa (Cowie et al. 2022).

The final three possible sources of bias concern underlying parasite and host population dynamics. First, parasites with multiple hosts in their life cycle (heteroxenous) are more vulnerable than parasites that require only one host (Farrell et al., 2021; Koh et al., 2004). For example, mites and digenean trematodes require multiple host species in their life cycle (chironomids, fish, or birds [Molloy et al., 1997]), which may also be endangered given the threatened state of freshwater ecosystems generally (e.g., Desforges et al., 2021). If any host in their life cycle goes extinct, they will too. This source of bias is especially difficult to account for given many trematode life cycles are yet to be appropriately characterized (Blasco‐Costa & Poulin, 2017). Second, parasites require a minimum host density to survive (Lafferty, 2004). Because of this, they will go extinct before their hosts do. Given that many critically endangered mussels survive only in single populations and that not all populations of mussels host every possible parasite of that species (Brian et al., 2021a), we argue it is likely that many parasites have already gone extinct from critically endangered mussel species. Third, we did not include facultative parasites or those with limited information as to their obligate nature, such as nematodes (McElwain et al., 2019), leeches (Bolotov et al., 2019), or chironomids and oligochaetes (Taskinen et al., 2021). In addition, we excluded bitterling fish (*Rhodeus amarus*), a parasite of European mussels, because this species can also be present in invasive molluscs, such as *Sinanodonta woodiana*. However, it could also be at risk from the loss of a large number of native freshwater mussel hosts. As such, the total diversity of mussel parasites is much higher than included here (see also Brian & Aldridge, 2019), and these excluded groups may also face extinction as freshwater mussels decline.

Therefore, while there is uncertainty around our estimates, we suggest that the total parasite richness of freshwater mussels in Europe and the United States and their corresponding extinction risk are likely higher than we estimated and that they should constitute a minimum estimate of how many species may be lost under current global trajectories.

### Comparisons with other estimates of parasite diversity and extinction risk

Our projections are commensurate with other predictions of undescribed diversity and extinction risk in parasitic organisms and reflect the disproportionately threatened nature of freshwater mussel hosts. Koh et al. (2004) estimated that 14% of “affiliates” (a broad range of commensals, mutualists, and parasites) were predicted to go extinct if their threatened hosts did, with more host‐specific affiliates the most at risk. These conclusions are similar to our 21% estimate. Farrell et al. (2015) found that generalist and specialist parasites are also differentially threatened by the loss of ungulate or carnivore hosts, though they did not estimate the proportion of threatened parasites explicitly. Regarding richness, Carlson et al. (2020a) recently predicted a total diversity of ∼100,000 helminth species (with 86% of trematodes undescribed), a more precise estimate than the 75,000–300,000 predicted by Dobson et al. (2008). Their estimate of 86% undescribed is similar to our 80% undescribed freshwater mussel trematodes.

These previous estimates occur over broad host and parasite scales (e.g., all vertebrates and all helminths), have correspondingly large margins of error, and only consider either richness or extinction risk (e.g., extinction risk is estimated using only currently described parasites). To our knowledge, we are the first to combine these two approaches in a specific host group and to consider all known obligate parasites, rather than just a specific taxonomic subset. As such, we provide some of the clearest evidence to date that parasites may be highly threatened and that increased attention must be paid to their diversity and conservation (Carlson et al., 2020b). Sixty parasite species of freshwater mussels are at immediate risk in Europe and the United States alone, many of which are not yet described. If all vulnerable mussels were to be lost, more ciliate and digenean trematode species would be lost than are currently described, lending weight to recent concerns that species could go extinct before we know of them (Lees & Pimm, 2015). Further, the predicted acceleration of parasite loss with host loss (Figure 3) emphasizes that trends in parasite generalism and specificity need to be understood in order to avoid underpredicting future losses based on current extinction trends. We strongly recommend a similar approach be taken across other host groups to build further evidence for the richness and extinction risk of parasites, especially for invertebrates that have been severely neglected in parasite studies (Brian & Aldridge, 2021; Wilson et al., 2015).

### Implications for the conservation of freshwater ecosystems

Although we believe there is moral value in conserving biodiversity for its own sake, a justification concerning the provision of useful ecosystem services is often demanded (Balvanera et al., 2001). In this respect, it is now widely accepted that parasites contribute to the functioning of freshwater ecosystems. Their free‐living biomass in transmission stages provides food for higher trophic levels (McKee et al., 2020; Mironova et al., 2020; Morley, 2012), and they are able to regulate host populations (Tompkins & Begon, 1999), facilitating the coexistence of multiple species (Strona, 2015). Specifically, sympatric freshwater mussel populations often host different parasite communities (e.g., Taskinen et al., 2021). Although the effects of freshwater mussel parasites are poorly understood, it is possible that host interspecific relationships are mediated to some extent by disparate parasite populations. We found that such interactions are not only poorly characterized but also at risk, with currently unknown consequences for freshwater mussel communities. As famously argued by Aldo Leopold, “To keep every cog and wheel is the first precaution of intelligent tinkering.” To stretch the metaphor, presently it is unknown what all the cogs and wheels are.

However, parasites can also negatively affect hosts at a variety of ecological scales. Both mites and trematodes can negatively affect the individual‐ and population‐level reproductive output of the freshwater mussel *Anodonta anatina* (Brian et al., 2021b), which could destabilize host populations (May & Anderson, 1978). Failing to account for this impact of parasitism could lead to overestimates of population fecundity based on current population size alone and thus underestimate the risk to threatened mussel species. Because mites can damage gill tissue (e.g., Fisher et al., 2000; Walker, 2017), which mussels use to filter water, parasites could also reduce the ecosystem services provided. Further, parasites significantly affect the filtration services provided by mussels, an effect that scales to the ecosystem level (Brian et al., 2022).

Finally, heteroxenous parasites can also have pronounced effects on other hosts (e.g., fish) in the life cycle, given the extremely high abundance they can reach in single individuals (e.g., over 900 metacercariae of the trematode *Rhipidocotyle fennica*, which uses mussels as its first intermediate host, were observed in a pike *Exos lucius* [Taskinen et al., 1991]). We suggest that the effect of both described and undescribed parasites is widespread and that the wider relationship between mussels and the freshwater environment cannot be appropriately understood without a greater emphasis on understanding their parasites.

We also highlight that certain geographic areas may be much more vulnerable to coextinctions than others. The currently described parasite diversity is much higher in the United States than in Europe. Our results suggest that this is due to the total greater diversity of mussel hosts there, rather than a greater number of parasite species per individual host. This supports the theory in parasite community ecology that host diversity begets parasite diversity (Johnson et al., 2016). That is, due to niche availability, a high number of possible host species will lead to parasite diversification and specialization, rather than a higher number of parasite species per host. This trend has been observed in the mites of freshwater mussels (Edwards & Vidrine, 2020). Given that highly diverse geographic areas also frequently have higher extinction risk, as is true for freshwater mussels (Lopes‐Lima et al., 2018), we recommend that much greater effort goes into characterizing the parasite diversity of biodiversity hotspots, as well as in general.

In sum, our results provide some of the most tangible evidence to date for the chronic underestimation of parasite richness and extinction risk, affirming the recent emphasis on characterizing parasite diversity and increasing efforts for their conservation (Carlson et al., 2020a, b; Dobson et al., 2008; Strona, 2015). Our conservative conclusions that 67% of freshwater mussel parasites in Europe and the United States are undescribed and that 21% are at risk of extinction have implications not only for estimates of total freshwater diversity but also for the structure and function of entire freshwater ecosystems.

## Supporting information

Supporting Information
Additional supporting information may be found in the online version of the article at the publisher's website.
