# Supplementary material for: Mussel parasite richness and risk of extinction
Source: Conserv Biol. 2022 Sep 26;36(6):e13979. doi: 10.1111/cobi.13979 (PMC10087751; doi:10.1111/cobi.13979)
Supplement: Supplementary file 1 — Supporting Information Additional supporting information may be found in the online version of the article at the publisher's website. [file COBI-36-0-s001.zip › cobi13979-sup-0001-Appendix_1.docx]

**Two thirds of freshwater mussel parasites are undescribed and over one fifth are at immediate risk of extinction: Appendix 1**

**Contents**

| Description of data files | 2 |
| --- | --- |
| Supplementary Tables | 4 |
| Supplementary Text | 6 |
| Supplementary References | 7 |

**Description of data files**

*.docx files*

Appendix 1 (THIS DOCUMENT)

- Lists all other appendices, and contains supplementary tables and text.

*R files*

Appendix 2

- Contains all required code to reproduce all analyses and figures presented in the manuscript
- All .csv files are used in this file

*.csv files*

Appendix 3

- Column 1 contains freshwater mussel species, and column 2 contains lists all aspidogastrean trematodes that are found in those host species

Appendix 4

- Column 1 contains freshwater mussel species, and column 2 contains lists all digenean trematodes that are found in those host species

Appendix 5

- Column 1 contains freshwater mussel species, and column 2 contains lists all mites that are found in those host species

Appendix 6

- Column 1 contains freshwater mussel species, and column 2 contains lists all aspidogastrean trematodes that are found in those host species

Appendix 7

- Each row is a parasite species. Column 1 describes how many host species it is found in (can be calculated independently from Data_S1 – Data_S4), column 2 describes how many total observations it has in those host species (calculated from supplementary tables of Brian & Aldridge 2019), and column 3 describes whether that parasite is from the USA or Europe.

Appendix 8

- Each row is a parasite species that is found in Europe. Column 1 describes what class of parasite it is, column 2 describes how many host species it is found in, and column 3 describes how many total observations it has in those host species.

Appendix 9

- Details as per ‘hostspecificityfocaleurope.csv’, but for parasites found in the USA rather than Europe.

*.xls files*

Appendix 10

- The first sheet provides a brief overview of the file, including how threat statuses were calculated.
- The second sheet consists of four columns. Column 1 lists all 295 host species in the database. Column 2 lists their location (USA or Europe). Column 3 lists their threat status according to the IUCN. Column 4 lists their threat status according to the US Fish and Wildlife Service.

**Supplementary Tables**

Table S1: Mean host range of aspidogastrean trematodes found in mussel hosts of different US Fish and Wildlife Service threat status. DD = Not listed or not threatened; TH = Threatened; EN = endangered. Dashes indicate that no hosts with that threat status were observed to have parasites. s.d. = standard deviation; n = the number of host species that the mean and s.d. estimates were calculated from.

|  | DD | TH | EN |
| --- | --- | --- | --- |
| Mean | 78.45 | 82.06 | 79.77 |
| s.d. | 12.44 | 12.54 | 15.50 |
| n | 91 | 6 | 10 |

Table S2: Mean host range of digenean trematodes found in mussel hosts of different US Fish and Wildlife Service threat status. DD = Not listed or not threatened; TH = Threatened; EN = endangered. Dashes indicate that no hosts with that threat status were observed to have parasites. s.d. = standard deviation; n = the number of host species that the mean and s.d. estimates were calculated from.

|  | DD | TH | EN |
| --- | --- | --- | --- |
| Mean | 4.15 | - | 4 |
| s.d. | 2.43 | - | 0 |
| n | 27 | - | 1 |

Table S3: Mean host range of mites found in mussel hosts of different US Fish and Wildlife Service threat status. DD = Not listed or not threatened; TH = Threatened; EN = endangered. Dashes indicate that no hosts with that threat status were observed to have parasites. s.d. = standard deviation; n = the number of host species that the mean and s.d. estimates were calculated from.

|  | DD | TH | EN |
| --- | --- | --- | --- |
| Mean | 9.02 | 3.67 | 4.6 |
| s.d. | 5.51 | 0.47 | 2.73 |
| n | 79 | 3 | 5 |

Table S4: Mean host range of ciliates found in mussel hosts of different US Fish and Wildlife Service threat status. DD = Not listed or not threatened; TH = Threatened; EN = endangered. Dashes indicate that no hosts with that threat status were observed to have parasites. s.d. = standard deviation; n = the number of host species that the mean and s.d. estimates were calculated from.

|  | DD | TH | EN |
| --- | --- | --- | --- |
| Mean | 14.22 | - | - |
| s.d. | 5.61 | - | - |
| n | 20 | - | - |

Table S5: Mean host range of aspidogastrean trematodes found in mussel hosts of different IUCN threat status. DD = data-deficient or not listed; LC = Least Concern; NT = Near Threatened; VU = Vulnerable; EN = endangered; CR = critically endangered. Dashes indicate that no hosts with that threat status were observed to have parasites. s.d. = standard deviation; n = the number of host species that the mean and s.d. estimates were calculated from.

|  | DD | LC | NT | VU | EN | CR |
| --- | --- | --- | --- | --- | --- | --- |
| Mean | 79.16 | 76.37 | 81.02 | 82.85 | 79.94 | 92 |
| s.d. | 10.25 | 13.16 | 13.09 | 13.70 | 14.27 | 0 |
| n | 27 | 45 | 17 | 8 | 8 | 2 |

Table S6: Mean host range of digenean trematodes found in mussel hosts of different IUCN threat status. DD = data-deficient or not listed; LC = Least Concern; NT = Near Threatened; VU = Vulnerable; EN = endangered; CR = critically endangered. Dashes indicate that no hosts with that threat status were observed to have parasites. s.d. = standard deviation; n = the number of host species that the mean and s.d. estimates were calculated from.

|  | DD | LC | NT | VU | EN | CR |
| --- | --- | --- | --- | --- | --- | --- |
| Mean | 4.98 | 3.68 | 7 | 2.25 | 4 | - |
| s.d. | 2.91 | 1.98 | 1 | 1.25 | 0 | - |
| n | 7 | 16 | 2 | 2 | 1 | - |

Table S7: Mean host range of mites found in mussel hosts of different IUCN threat status. DD = data-deficient or not listed; LC = Least Concern; NT = Near Threatened; VU = Vulnerable; EN = endangered; CR = critically endangered. Dashes indicate that no hosts with that threat status were observed to have parasites. s.d. = standard deviation; n = the number of host species that the mean and s.d. estimates were calculated from.

|  | DD | LC | NT | VU | EN | CR |
| --- | --- | --- | --- | --- | --- | --- |
| Mean | 9.36 | 8.25 | 8.57 | 7.40 | 8.30 | - |
| s.d. | 5.55 | 5.34 | 6.30 | 2.42 | 5.64 | - |
| n | 26 | 39 | 12 | 5 | 5 | - |

Table S8: Mean host range of ciliates found in mussel hosts of different IUCN threat status. DD = data-deficient or not listed; LC = Least Concern; NT = Near Threatened; VU = Vulnerable; EN = endangered; CR = critically endangered. Dashes indicate that no hosts with that threat status were observed to have parasites. s.d. = standard deviation; n = the number of host species that the mean and s.d. estimates were calculated from.

|  | DD | LC | NT | VU | EN | CR |
| --- | --- | --- | --- | --- | --- | --- |
| Mean | 14.6 | 14.06 | - | 14.25 | - | - |
| s.d. | 6.8 | 5.33 | - | 3.75 | - | - |
| n | 5 | 13 | - | 2 | - | - |

Table S9: Percentage of parasite species predicted to go extinct if all critically endangered (CR) mussel hosts go extinct, if all critically endangered and endangered (CR + EN) hosts go extinct, and if all critically endangered, endangered and vulnerable (CR + EN + VU) hosts go extinct.

| Hosts extinct | Aspidogastreans extinct | Digeneans extinct | Mites extinct | Ciliates extinct | Total parasites extinct |
| --- | --- | --- | --- | --- | --- |
| CR | 2.9% | 11.3% | 8.8% | 10.2% | **9.3%** |
| CR + EN | 5.3% | 19.9% | 15.6% | 18.0% | **17.0%** |
| CR + EN + VU | 6.8% | 25.1% | 19.9% | 22.8% | **21.8%** |

**Supplementary Text**

It has been suggested that threatened species have, on average, fewer specialist parasite species than non-threatened hosts, and fewer parasites in general. This could be due to long-term evolutionary dynamics; for example, specialised parasites are far more likely to persist if they target common hosts (Strona et al. 2013). It could also be due to the fact that, given parasites require a certain host density to successfully transmit and persist in the population (Lafferty 2004), threatened species have *already* had a proportion of their parasites go extinct, thanks to a terminal reduction in the density of their hosts (Altizer et al. 2007). The two explanations are also not mutually exclusive. In either case, if this pattern holds, then parasites would be at a lower risk of extinction than predicted by Fig. 3 (main text), as the species most likely to go extinct first (i.e. threatened mussels) will have a lower than predicted share of the parasites.

However, it does not appear that this is the case for freshwater mussels. If it were true that threatened hosts had fewer specialist parasites than non-threatened hosts, then the average host range of parasites found in TH or EN mussels (CR, EN and VU mussels respectively) should be larger than the average host range of parasites found in DD mussels (DD, LC and NT mussels respectively). In other words, non-threatened mussels should have more specific parasites. Looking across Tables S1 – S8, there is no evidence that this is the case, with the mean host ranges of parasites in threatened hosts being comparable to those in non-threatened hosts. Indeed, for digenean trematodes (Table S2, S6) and mites (Table S3, S7), the overall mean host range of parasites found in VU and EN hosts is smaller than in LC and NT hosts. Therefore, in the absence of other clear evidence it seems reasonable to assert than the loss of threatened freshwater mussel hosts will impact parasite diversity as much as the loss of non-threatened hosts. This may have occurred because many endangered freshwater mussel populations may have experienced dramatic recent declines (Bogan et al. 1993), rather than being inherently rare, and thus parasites do not show the expected evolutionary response to host rarity.

The comparison between threatened and non-threatened hosts is also hampered by varying sample sizes: note that the number of non-threatened hosts sampled in Tables S1 – S8 is significantly higher than non-threatened hosts. This pattern has been previously recognised and discussed in greater detail (see Brian & Aldridge 2019), but it again emphasises the dearth of sampling and lack of understanding of freshwater mussel parasite diversity, especially in the most endangered hosts where the impacts of parasitism may be disproportionately high.

**Supplementary References**

Altizer, S., Nunn, C. L., & Lindenfors, P. (2007). Do threatened hosts have fewer parasites? A comparative study in primates. *Journal of Animal Ecology*, **76**(2), 304-314.

Bogan, A. E. (1993). Freshwater bivalve extinctions (Mollusca: Unionoida): a search for causes. *American Zoologist*, **33**(6), 599-609.

Brian, J. I., & Aldridge, D. C. (2019). Endosymbionts: An overlooked threat in the conservation of freshwater mussels?. *Biological Conservation*, **237**, 155-165.

Lafferty, K. D. (2004). Fishing for lobsters indirectly increases epidemics in sea urchins. *Ecological Applications*, **14**(5), 1566-1573.

Strona, G., Galli, P., & Fattorini, S. (2013). Fish parasites resolve the paradox of missing coextinctions. *Nature Communications*, **4**(1), 1-5.
